# Evaluating the Analgesic Efficacy of Superficial Cervical Plexus Block for Head and Neck Surgeries: A Comparative Randomized Control Study

**DOI:** 10.7759/cureus.39303

**Published:** 2023-05-21

**Authors:** Himani Patel, Neha Shah, Afroza Syed, Panjari Shah, Sharmy Macwan

**Affiliations:** 1 Department of Anaesthesia, Government Medical College, Sir Sayajirao General Hospital, Vadodara, IND

**Keywords:** perioperative, superiority, scpb, landmarks, head and neck region

## Abstract

Introduction: In the present study, the advantages of superficial cervical plexus block (SCPB) were evaluated using a landmark-guided method. Our primary aim was to evaluate the analgesic efficacy of SCPB in various head and neck surgeries by observing intra- and postoperative requirements of the total dose of the systemic analgesic, visual analog scale (VAS) score, and the total duration of analgesia; vital parameters and associated perioperative complications were also observed.

Materials and methods: Sample size was calculated using reference by taking the parameter pain score at 12 hours using MedCalc software v. 19.5.1 (MedCalc Software, Ostend, Belgium) with a mean difference of score 3±3.5 between two groups, 80% power, and 95% confidence interval (CI); the sample size for each group was 21. There were 30 patients in each group of ASA I, II, and III who were posted for mandibular, tympanomastoid and clavicular surgeries. Group A received general anaesthesia with systemic analgesia and Group B received general anaesthesia followed by SCPB with an injection of bupivacaine 0.25% 10ml on each side according to the site of surgery. VAS score, intra and postoperative analgesic requirement in 24 hours, time of first demand bolus, and peri-operative complications were noted.

Results: Intraoperative fentanyl requirement for group A was 97.5±13.75 µg as compared to group B (70.16±13.09 µg), postoperative injection paracetamol requirement was also significantly higher in group A (2566.66±504 mg) as compared to group B (833.33±874.28 mg). The total duration of analgesia was significantly higher in Group B (1191.33±375.36 min) as compared to Group A (122.0±50.88 min) with a p-value <0.0001. No significant complications were noted in any patient.

Conclusion: SCPB provides better perioperative analgesia by decreasing intraoperative as well as postoperative systemic analgesic requirements and their associated side effects, with no significant perioperative complications in various head and neck region surgeries.

## Introduction

The superficial cervical plexus (SCP) arises from the anterior rami of the C1-C4 spinal nerves and forms four cutaneous branches: greater auricular, lesser occipital, transverse cervical and supraclavicular nerves, which contribute sensory innervation to the cutaneous structures of the anterolateral portion of the neck and external ear and tip of the shoulder [1]. An SCP block involves the administration of subcutaneous injection at the centre of the posterior margin of the sternocleidomastoid (SCM) muscle aiming at the cutaneous branches of the cervical plexus, which is usually executed by adopting ultrasound guidance or anatomical landmarks. A deep cervical plexus block (CPB) involves placing the needle between the prevertebral layer of fascia and the cervical nerve roots at the level of C2-C4 while targeting the superficial and deep branches of the cervical plexus concurrently. Despite a few benefits, deep CPB may require a thorough risk-benefit analysis prior to its deployment though it is a routinely performed procedure via ultrasound guidance (USG) [2,3].

In 2004, Telford and Stoneham proposed the term ‘intermediate cervical plexus blockade’ involving local anaesthetic injection beneath the investing layer based on the study by Pandit et al. on cadavers [3-4]. In 2010, Choquet et al. attempted to reframe the idea of intermediate CPB under USG. They placed an injecting needle into the posterior cervical space (between the SCM muscle and prevertebral layer) at the C4 level using USG, focusing the superficial branches of the cervical plexus together with sensory or both sensory-motor branches of the cervical plexus presumptively, supplying the SCM muscle [5]. The landmark-guided technique is easy to perform but determining the needle’s position can pose a challenge. Furthermore, ‘superficial, intermediate and deep’ are not well-defined anatomical terminologies that can describe the topographic relations between the tissue and skin sufficiently [2,3]. Superficial CPB (SCPB) can be given for analgesia after various head and neck surgeries such as mandibular, tympanomastoid, thyroid, submandibular, clavicular and various other neck surgeries. SCPB can also be used as an independent anaesthetic technique for exterior ear surgery [2].

We aimed to evaluate the analgesic effect of landmark-guided SCPB in mandibular, tympanomastoid and clavicular surgeries by comparing it with conventional general anaesthesia using systemic analgesic through observation of intra- and postoperative requirements of the total dose of systemic analgesics, visual analog scale (VAS) score, total duration of analgesia, vital parameters and the occurrence of any perioperative complications.

## Materials and methods

This study was carried out in the Department of Anesthesiology, Government Medical College and Sir Sayajirao General Hospital, Vadodara, from June 2021 to December 2021. The study protocol of this prospective randomized clinical study was approved by the Institutional Ethics Committee for Biomedical and Health Research (IECBHR), Government Medical College and SSG Hospital, Baroda. The CTRI registration no. for the same is CTRI/2021/06/034429.

The sample size was calculated using reference standards, by taking the parameter pain score at 12 hours, using MedCalc 19.5.1. version (MedCalc Software, Ostend, Belgium), with a mean difference of score 3±3.5 between the two groups, 80% power, 95% confidence interval (CI), alpha error of 5% and beta error of 20%. The sample size for each group was 21. We enrolled 30 patients in each group [6].

After a thorough pre-anaesthetic check-up, written informed consent was obtained from all the patients. Inclusion criteria were patients between 18-70 years of age of either sex, 40-75 kg weight, ASA grade I/II/III, having undergone duration of surgery ≤ 3 hours, posted for mandibular, tympanomastoid and clavicular surgery, able to give written and informed consent, and able to understand VAS regarding the assessment of pain. Patients with coagulopathy, local infection, pre-existing neuropathy or any other neurological disease, hypersensitivity to local anaesthetic drugs and psychiatric illness were excluded from the study. The study population were randomly allocated to two groups of 30 patients each, using the sealed envelope method. All the patients received general anaesthesia.

Baseline vitals were noted for all the participants, and they all received premedication in the form of intravenous injection (inj.) of glycopyrrolate 0.2 milligrams (mg), inj. of ondansetron 4 mg, inj. of midazolam 1 mg, inj. of paracetamol (PCM) 1000 mg, and inj. of fentanyl 1 microgram/kilogram (µg/kg). General anaesthesia (GA) was induced with intravenous inj. of propofol 2-2.5 mg/kg, inj. of suxamethonium chloride 1.5-2 mg/kg and inj. of vecuronium bromide 0.1 mg/kg was given for muscle relaxation after intubation. In Group B patients, superficial CPB was administered in a supine position with the head turned opposite to the side of the block.

SCPB technique

**Figure 1:**
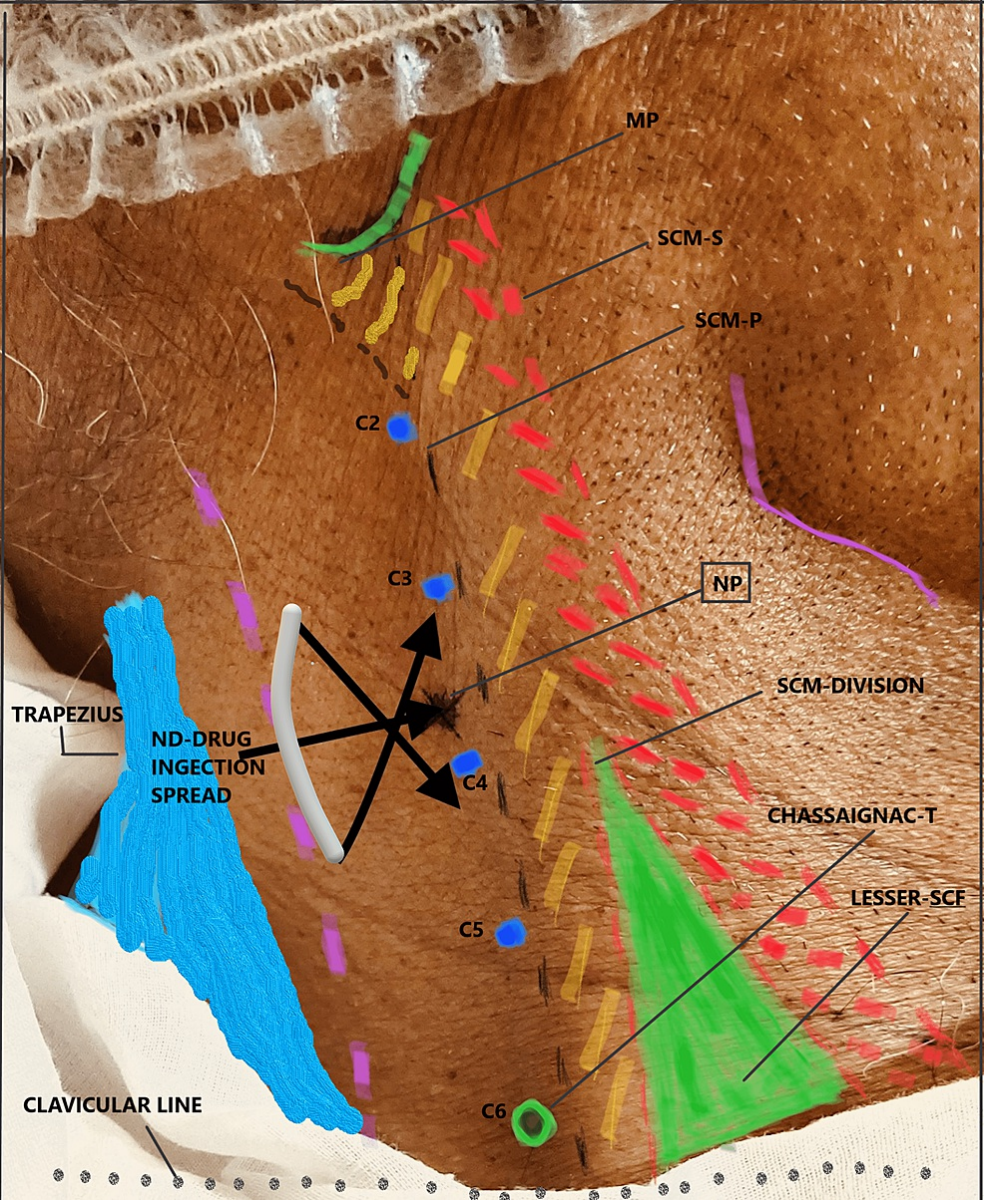
Landmarks for the superficial cervical plexus block. MP: mastoid process; SCM: sternocleidomastoid muscle; SCM-S: SCM sternal border; SCM-P: SCM posterior border; NP: needle entry point where the drug was injected; Chassaignac-T: Chassaignac tubercle; lesser-SCF: lesser supraclavicular fossa; C2-C6 (Chassaignac tubercle) are transverse processes of cervical vertebrae; ND: needle direction for drug injection in caudad and cephalad direction along the posterior border of SCM.

Anatomical landmarks were identified and marked as shown in Figure 1. The site of needle insertion was marked at the midpoint of the posterior border of the sternocleidomastoid, a line that is drawn extending from the mastoid process to Chassaignac’s tubercle (the transverse process of the sixth cervical vertebra). At this point, the branches of the superficial cervical plexus emerge behind the posterior border of the SCM muscle. Local anaesthetic was injected alongside the posterior border of the muscle 2-3 cm in cranial and caudal directions. Using a ‘fan’ technique, the local anaesthetic was administered subcutaneously and behind the sternocleidomastoid muscle. Ten millilitres of 0.25% bupivacaine were injected for each side of the block using a 1.5-inch 24-gauge needle [7].

General anaesthesia was maintained with O2 + air (50:50), 3-litre flow, sevoflurane 2% to 2.5% and an intravenous inj. of vecuronium bromide 0.025 mg/kg. An intraoperative rise in heart rate (>100/min) and systolic blood pressure (≥ 20% from pre-induction value) were managed by giving intravenous inj. of fentanyl 0.5 microgram/kg in incremental doses (dose of fentanyl did not exceed 3 µg/kg for less than three hours of surgery). At the end of the surgery, the reversal of residual neuromuscular block was done and the patient was extubated. The patient was then transferred to the recovery room and the severity of pain was assessed by a VAS, which is a straight line denoting one end with no pain and the other end with the worst pain. VAS score was calculated as follows: 0, no pain; 1-3, mild pain; 4-6, moderate pain; 7-9, severe pain; 10, worst pain. The VAS score was assessed when the patient could follow verbal commands. 

Evaluation of block for adequate analgesia intraoperatively was done in terms of the total requirement of intravenous analgesics through the observation of heart rate and blood pressure and postoperatively, it was done by observing VAS score, time of first demand for a bolus of rescue analgesic (intravenous paracetamol) and total requirement of rescue analgesic in 24 hours. The total duration of analgesia was considered from the time of incision until the first demand bolus of rescue analgesia postoperatively. Pulse, mean blood pressure (BP), oxygen saturation (SpO2) and any side effects were also noted. 

Statistical analysis of the quantitative data for various parameters was done using the Student's t-test and the chi-square test was used for qualitative (nonparametric) data using MedCalc software version 19.5.1. Observed data were presented in mean ± standard deviation (SD). The significance of the statistical analysis was judged by the p-value. P ˃ 0.05 was considered nonsignificant, p < 0.05 was considered significant and p < 0.01 was considered highly significant.

## Results

As shown in Table 1, demographic data were comparable between Group A and Group B. 

**Table 1:** Demographic data SD: standard deviation

Parameter	Group A	Group B	Intergroup p-value
Number of patients	30	30	˃ 0.05
Age (Years) (Mean ± SD)	31.86 ± 6.79	32.06 ± 9.48	˃ 0.05
Sex (Male : Female)	26 : 4	24 : 6	> 0.05
Weight (Kg) (Mean ± SD)	57.66 ± 6.53	58.5 ± 6.31	˃ 0.05
ASA I	6 (20 %)	5 (16.66 %)	> 0.05
ASA II	19 (63.33 %)	18 (60 %)	˃ 0.05
ASA III	5 (16.66 %)	7 (23.33 %)	˃ 0.05
Duration of Surgery (Minutes)	89.66 ± 43.03	99.33 ± 38.76	˃ 0.05

**Figure 2:**
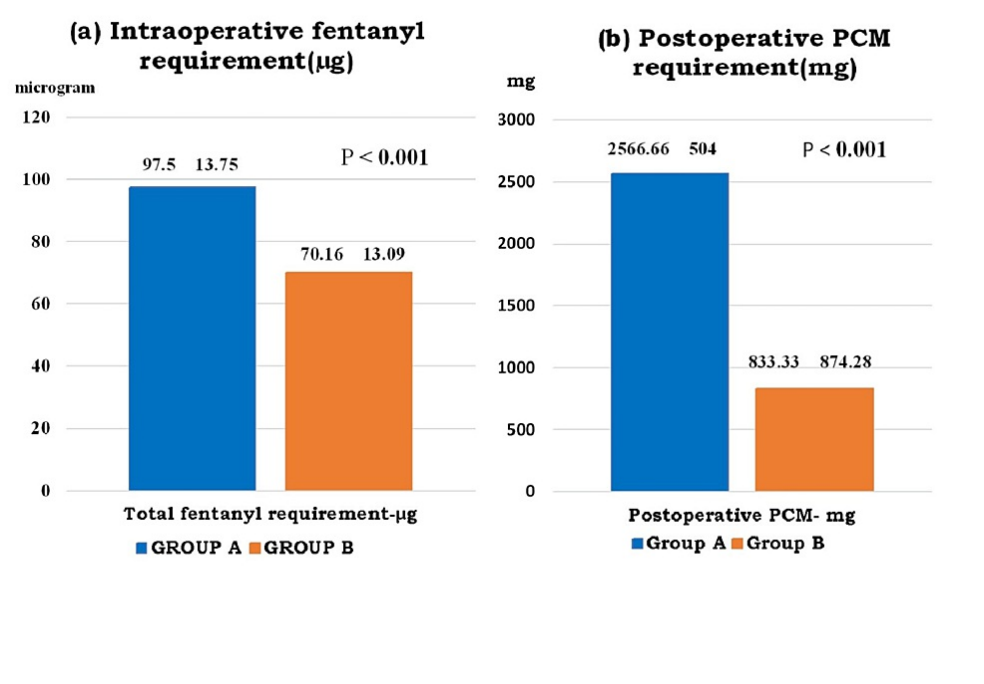
(a) Intraoperative total fentanyl requirement (b) postoperative total rescue analgesic requirement PCM: paracetamol, µg: microgram, mg: milligram

Figure 2 shows that the intraoperative total fentanyl requirement and the postoperative rescue analgesic (inj. of paracetamol 1gm) requirement in 24 hours were significantly higher in Group A (97.5±13.75 µg and 2566.66±504 mg) as compared to Group B (70.16±13.09 µg and 833.33±874.28 mg); p-value was < 0.001. 

**Figure 3:**
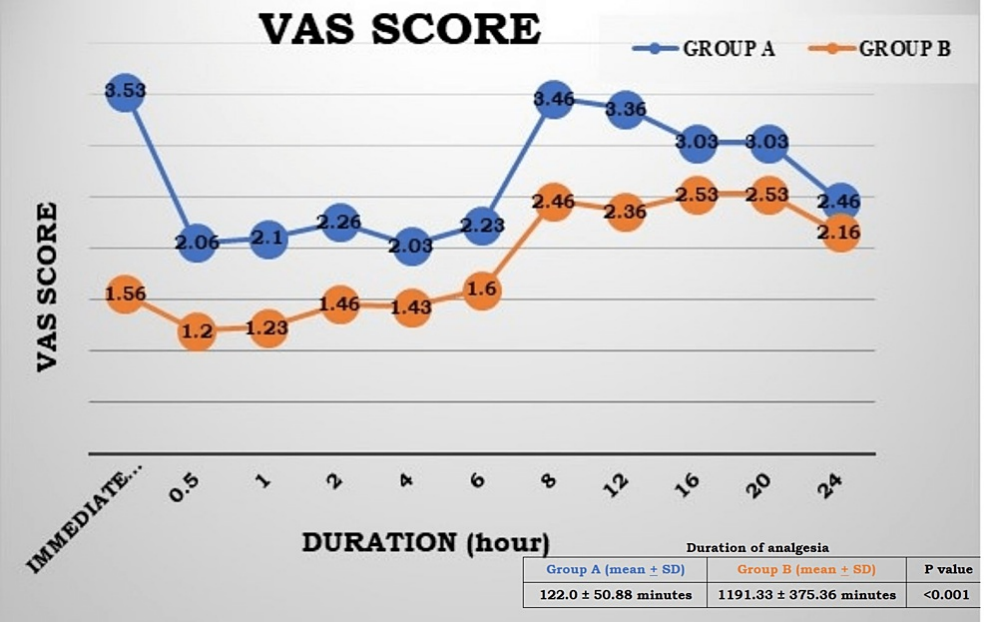
Postoperative mean VAS score and duration of analgesia VAS: visual analogue scale

As shown in Figure 3, the mean VAS score was significantly lower in Group B during the postoperative period from immediate postoperative to 12 hours as compared to Group A (p-value <0.05). The difference was insignificant at 16, 20 and 24 hours, with a p-value >0.05. The total duration of analgesia was also significantly higher in Group B (block group) as compared to Group A.

Table 2 shows that 23 patients in Group A had VAS ≥4 whereas none of the patients in Group B had VAS ≥4 within half an hour, including in the immediate postoperative period. At six hours also none of the patients had reported VAS ≥4; the observed effect was due to the administration of rescue analgesics or the effect of the block. 

**Table 2:** Number of patients in each group with postoperative VAS score ≥ 4 in different types of surgeries VAS: visual analog scale; PACU: post-anaesthesia care unit

Type of Surgery	Groups	Immediately (In PACU)	1/2 hour	6 hours	8 hours	12 hours
Mandibular	A	9	4	0	7	13
	B	0	0	0	0	9
Clavicular	A	5	0	0	4	1
	B	0	0	0	0	0
Tympanomastoid	A	5	0	0	4	1
	B	0	0	0	4	1

Tables 3-4 show intra- and postoperative changes in pulse rate, mean arterial pressure and SpO2. No significant difference was observed in hemodynamics and changes remained within the clinically normal ranges. Other parameters such as SpO2 and respiratory rate also remained stable. 

**Table 3:** Intraoperative changes in mean pulse rate, MAP, SpO2 and EtCO2 MAP: mean arterial pressure, SpO2: oxygen saturation, EtCO2: end-tidal carbon dioxide, SD: standard deviation, immediate P: immediate postoperative period means five minutes after extubation

Time (minutes)	Pulse rate (mean ± SD)			MAP (mean ± SD)			SpO2 % (mean ± SD)			EtCO2 mmHg (mean ± SD)		
	Group A	Group B	P-value	Group A	Group B	P-value	Group A	Group B	P-value	Group A	Group B	P-value
At baseline	103 ± 8.23	108 ± 5.78	> 0.05	100.11 ± 4.00	102.4 ± 5.30	> 0.05	98.13 ± 0.25	99.13 ± 0.57	>0.05	33.06 ± 1.14	32.96 ± 1.14	>0.05
After incision	92.46 ± 17.49	92.2 ± 7.93	> 0.05	84.42 ± 6.08	84.15 ± 5.01	> 0.05	98.96 ± 0.18	99.3 ± 0.46	>0.05	33.06 ± 1.14	33 ± 1.14	>0.05
5	93.93 ± 18.40	86.66 ± 9.56	> 0.05	89.62 ± 10.2	84.88 ± 5.03	> 0.05	-	-	-	-	-	-
10	89.2 ± 13.09	81.73 ± 7.51	> 0.05	86.02 ± 6.53	81.75 ± 4.05	> 0.05	-	-	-	-	-	-
20	89.13 ± 15.39	82.33 ± 10.91	> 0.05	86.44 ± 8.60	84.82 ± 5.95	> 0.05	-	-	-	-	-	-
30	80.6 ± 5.33	79.8 ± 4.27	> 0.05	81.6 ± 4.37	84.51 ± 4.27	> 0.05	99.5 ± 0.50	99.46 ± 0.50	>0.05	33.06 ± 1.14	32.26 ± 0.98	>0.05
60	81.91 ± 8.03	76.61 ± 4.55	> 0.05	82.49 ± 7.23	82.92 ± 3.17	> 0.05	99.5 ± 0.50	99.4 ± 0.49	>0.05	33 ± 1.14	32.93 ± 1.14	>0.05
90	81.4 ± 4.42	79.75 ± 3.76	> 0.05	85.2 ± 4.76	82.05 ± 3.14	> 0.05	99.36 ± 0.66	99.46 ± 0.50	>0.05	33.06 ± 1.14	33.06 ± 1.14	>0.05
20	83.66 ± 3.44	86.4 ± 1.67	> 0.05	85.56 ± 2.45	83.46 ± 2.18	> 0.05	99.36 ± 0.49	99.46 ± 0.50	>0.05	32.93 ± 1.14	33.06 ± 1.25	>0.05
150	81 ± 2.75	84 ± 2.0	> 0.05	84.56 ± 2.99	82.13 ± 2.72	> 0.05	99.46 ± 0.50	99.5 ± 0.57	>0.05	33.06 ± 1.15	33.26 ± 1.14	>0.05
180	86 ± 3.16	84 ± 2.0	> 0.05	86.53 ± 2.32	84.44 ± 4.01	> 0.05	99.35 ± 0.48	99.0 ± 0.62	>0.05	33.07 ± 1.15	33.18 ± 1.27	>0.05

**Table 4:** Postoperative changes in mean pulse rate, MAP and SpO2 MAP: mean arterial pressure, SpO2: oxygen saturation, SD: standard deviation, immediate P: immediate postoperative period means five minutes after extubation.

Duration (hours)	Pulse rate (mean ± SD)			MAP (mean ± SD)			SpO2 % (mean ± SD)	
	Group A	Group B	P-value	Group A	Group B	P-Value	Group B	P-value
Immediate p.	103.93 ± 16.47	102.93 ± 7.94	>0.05	100.42 ± 7.43	99.2 ± 3.48	>0.05	99.4 ± 0.56	>0.05
½	86.93 ± 9.31	83.46 ± 7.94	>0.05	93.24 ± 10.75	95.02 ± 3.65	>0.05	99.5 ± 0.62	>0.05
1	82.86 ± 13.82	81.13 ± 5.91	>0.05	93.11 ± 4.54	93.91 ± 3.7	>0.05	99.4 ± 0.49	>0.05
2	86.86 ± 14.61	78.26 ± 4.68	>0.05	92.46 ± 10.77	92.42 ± 3.63	>0.05	99.56 ± 0.50	>0.05
4	78.86 ± 4.86	80.66 ± 3.72	>0.05	92.44 ± 3.73	94.17 ± 3.02	>0.05	99.53 ± 0.57	>0.05
6	80.6 ± 3.6	81.86 ± 7.02	>0.05	93.08 ± 2.55	95.2 ± 3.07	>0.05	99.53 ± 0.50	>0.05
8	91.6 ± 13.6	84.86 ±11.57	>0.05	95.13 ± 4.28	95.26 ± 3.5	>0.05	99.5 ± 0.57	>0.05
12	96.26 ± 16.12	84.13 ± 9.14	>0.05	95.91 ± 3.35	95.04 ± 3.56	>0.05	99.5 ± 0.62	>0.05
16	91.8 ± 14.59	84.8 ± 8.59	>0.05	95.33 ± 4.58	95.88 ± 2.37	>0.05	99.4 ± 0.62	>0.05
20	90.46 ±14.09	83.93 ±5.74	>0.05	96.22 ± 3.14	94.15 ± 2.37	>0.05	99.36 ± 0.66	>0.05
24	82.43 ±4.42	81.26± 3.76	>0.05	94.55 ± 3.82	94.51 ± 3.11	>0.05	99.56 ± 0.56	>0.05

No serious perioperative complications were observed in either group except for two cases of postoperative nausea and vomiting (PONV) in Group A who underwent tympanomastoid surgery (Table 5).

**Table 5:** Incidence of perioperative complications PONV: Postoperative nausea and vomiting

Perioperative complications	Group A (n=30)	Group B (n=30)
Respiratory depression	0	0
Local Anaesthetic toxicity/Intravascular injection	0	0
Hypersensitivity	0	0
Hematoma/infection at site of injection	0	0
Horner’s syndrome	0	0
PONV	2 (6.66 %)	0
Vertigo	0	0

## Discussion

In the present study, the effect of SCPB was primarily evaluated, and it was observed that the intraoperative total fentanyl requirement was significantly higher in Group A (97.5±13.75 µg) than in Group B (70.16±13.09 µg) with a p-value <0.001, postoperative rescue analgesia (paracetamol) requirement was also significantly higher in Group A (2566.66±504 mg) than in Group B (833.33±874.28 mg) with p-value <0.001, postoperative mean VAS score was significantly lower in Group B than in Group A up to 12 hours with p-value <0.05 and total duration of analgesia was significantly higher in Group B (1191.33±375.36 min) as compared to Group A (122.0±50.88 min) with a p-value <0.001. It indicates that SCPB provides good postoperative analgesia. Secondarily, no significant difference was observed in hemodynamic parameters, and also no serious perioperative complications were present in either group except in two patients who underwent tympanomastoid surgery in Group A who had PONV in the postoperative period.

The superficial CPB is a routinely performed subcutaneous injection technique at the centre of the posterior margin of the SCM muscle targeting cutaneous branches of the cervical plexus [3]. Nonetheless, in 2003, Pandit et al. used a novel sub-investing fascial injection technique (superficial to the prevertebral fascia but underneath the investing fascia) for superficial CPB. They compared superficial and intermediate cervical plexus blocks and concluded a comparable analgesic effect in patients posted for carotid endarterectomy. Furthermore, they considered the intermediate block as a part of the superficial cervical plexus block. Pandit et al. further explained that there is probable communication between the superficial and deep spaces through the prevertebral fascia, which could explain why the efficacy of the superficial CPB is comparable to that of the deep CPB and combined deep and superficial CPB during carotid endarterectomy [3,8]. Pandit et al. also conducted a systemic review to assess the complication rate associated with superficial and deep CPB and reported that superficial/intermediate block is relatively safer than deep block [9].

We have observed that intraoperative total fentanyl requirement and postoperative rescue analgesia (paracetamol) requirement were significantly higher in Group A as compared to Group B. Postoperative mean VAS score was significantly lower in Group B than in Group A up to 12 hours. Similarly, Aweke et al. studied bilateral SCPB in thyroidectomy patients and reported that SCPB significantly decrease the median postoperative pain score (NRS: numerical rating scale), which was 3 in the block group and 5 in the control group (p = 0.002). They also reported considerable differences at the 6th, 12th and 24th hours showing lower median pain scores in the SCPB group compared to the control group. They reported that the total analgesia requirement in the form of median tramadol consumption within 24 hours was zero and the time until the first analgesic request was also prolonged in the block group [10]. We found that the total duration of analgesia was significantly higher in Group B (1191.33±375.36 minutes) as compared to Group A (122.0±50.88 minutes); p-value <0.001. Similarly, Aweke et al. found that the time for first rescue analgesia was significantly earlier in the control group than in the block group [10]. Improved perioperative analgesia with a lower pain score in the block group following thyroid surgery was also reported by Aweke et al. and Andrieu et al [10,11].

As shown in Table 2, most of the patients in Group A had VAS ≥4 within half an hour in the immediate postoperative period and were given rescue analgesics in the form of inj. paracetamol 1000 mg IV., whereas none of the patients in Group B had VAS ≥4 during the immediate postoperative period. At six hours none of the patients had VAS ≥4 owing to the administration of rescue analgesics in both groups or the observed effect was estimated to be due to block. In the present study, we found that in Group A seven out of 20 patients posted for mandibular surgery had VAS ≥4 at 8 hours, compared to zero patients in Group B. At 12 hours, postoperatively 13 patients in Group A were compared to nine patients in Group B who had VAS ≥4 and were given rescue analgesia.

Hakim et al. used SCPB for analgesia for treating infections in peri-mandibular spaces which comprised abscesses in the deeper facial spaces, submandibular and cervical lesions requiring dissection in the deeper planes and fractures of mandibular angle region. They combined SCP block with conventional nerve blocks such as the inferior alveolar and long buccal nerve blocks, which led to optimal anaesthesia and positive outcomes. A similar result was reported by Kanthan et al. in their study [12,13]. Other researchers also studied the effect of SCPB in perimandibular and fasciomaxillary surgeries and found SCPB to be more effective [14-16].

In clavicular surgeries, four out of five patients in Group A had VAS ≥4 at eight hours while one patient had VAS ≥4 at 12 hours, whereas none of the patients in Group B had VAS ≥4 up to 12 hours in our study. Similarly, Banerjee and colleagues also reported low pain scores among the patients with the use of SCPB. They also reported the advantage of USG-guided interscalene + SCPB for anaesthesia in patients posted for clavicular surgery and reported longer pain-free periods, lesser opioid requirements and shorter PACU stays in patients receiving blocks compared to patients who received general anaesthesia. They concluded that interscalene + SCP block is a safe and effective mode of anaesthesia for patients undergoing clavicular surgery [17]. Few other studies also found the beneficial effect of SCPB in clavicular surgeries [18,19].

We observed that in tympanomastoid surgery of Group A, the first dose of rescue analgesics was required immediately postoperatively in all the patients. Four patients in Group A required a second dose of systemic analgesic at eight hours and another patient required a second dose at 12 hours, whereas four patients in Group B required the first dose of rescue analgesic at eight hours and another patient required the same at the 12th hour. Thus, the effect duration of analgesia is relatively shorter in tympanomastoid surgeries; however, to confirm the same, a larger study cohort is required. Similarly, Deepika et al. studied the effect of SCPB on patients with total mastoidectomy and found postoperatively lower VAS scores and decreased requirement of systemic analgesics in the block group compared to the non-block group [20]. As a part of multimodal analgesia, the advantage of SCPB was mentioned by other authors also in their studies on patients of mastoidectomy [21-24].

We did not observe any significant hemodynamic difference between the two groups. An intraoperative transient rise in pulse rate and blood pressure was managed by giving analgesia in the form of an incremental dose of IV fentanyl and concurrently ensuring adequate depth of anaesthesia in both groups by maintaining recommended dial concentration of sevoflurane and constant fresh gas flow throughout the surgery. Although in three tympanomastoid patients of Group A, even after administering the recommended analgesia and ensuring adequate anaesthesia depth, we observed a blood pressure raise pattern which was then controlled by initiating a nitroglycerine (5 µg/kg/min dose) drip. This was interpreted to be due to inadequate pain control by systemic analgesics during deep drilling in such patients. We did not observe a such problem in Group B, hence we state that SCPB provides good analgesia in tympanomastoid patients.

In two patients who underwent tympanomastoid surgery, PONV was reported. Although, along with fentanyl, we also must consider surgical aetiology in such patients as all the patients were given antiemetics in the form of intravenous ondansetron.

Limitations

We included a variety of head and neck surgeries in our study owing to the COVID-19 pandemic, as the average number of elective surgeries performed at that time was low. But to minimize this confounder effect, we considered the same number of each surgery type in both groups. We also could not practice observer blinding in this study due to limited working staff in the operation theatre. We used the landmark guided technique for block and recorded optimum outcomes with this technique. We have not utilized USG machines for the unavailability of these machines equally across all theatres. Thus, we used the landmark-guided easy-to-perform technique for this study.

## Conclusions

We conclude that landmark-guided superficial cervical plexus block is easy to perform and can be routinely used as a part of a balanced anaesthesia technique during several head and neck surgeries. It provides superior pain relief concerning intraoperative analgesia and postoperative analgesia. It also delivers an extended duration of analgesia without detrimental perioperative complications.
